# Non-Parametric Bayesian Human Motion Recognition Using a Single MEMS Tri-Axial Accelerometer

**DOI:** 10.3390/s121013185

**Published:** 2012-09-27

**Authors:** M. Ejaz Ahmed, Ju Bin Song

**Affiliations:** Department of Electronics and Radio Engineering, Kyung Hee University, Yongin 446-701, Korea; E-Mail: ejaz629@gmail.com

**Keywords:** MEMS application, human motion recognition, non-parametric Bayesian inference, infinite Gaussian mixture model, Gibbs sampler

## Abstract

In this paper, we propose a non-parametric clustering method to recognize the number of human motions using features which are obtained from a single microelectromechanical system (MEMS) accelerometer. Since the number of human motions under consideration is not known *a priori* and because of the unsupervised nature of the proposed technique, there is no need to collect training data for the human motions. The infinite Gaussian mixture model (IGMM) and collapsed Gibbs sampler are adopted to cluster the human motions using extracted features. From the experimental results, we show that the unanticipated human motions are detected and recognized with significant accuracy, as compared with the parametric Fuzzy C-Mean (FCM) technique, the unsupervised K-means algorithm, and the non-parametric mean-shift method.

## 1. Introduction

Human motion recognition (HMR) is an important topic currently being researched due to its large number of applications in tracking, personal navigation, health care, personal life log, surveillance, and sports, among other things. Human motion recognition [1–13] systems have been proposed to efficiently detect numerous human motions, and they are widely applicable in the above mentioned domains. However, the motion recognition system has some limitations that cannot be mitigated. One of these limitations is inherent in the dynamic nature of human motions; that is, the daily motions of a person are not limited but rather vary from a small to a large set of motions. The introduction of new human motions makes detection and recognition challenging. Therefore, there is a need for a HMR system that is adaptable, accurate, and robust to the dynamic nature of the daily motions performed by a human.

In the health care domain, motions of patients are monitored via wearable sensors. This is useful for three reasons: (1) to keep track of the movements performed by a patient during the medical examination period, (2) to reduce the number of patient visits to medical facilities, and (3) automated motions retrieval and management to facilitate the documentation of a patient's history.

Two main technologies being currently used for motion recognition are body-mounted sensors (accelerometers) [1,2] or image-based recognition (cameras) [3,5] for monitoring a subject's motions. Both methodologies have their own pros and cons. The benefit of camera aided motion identification is that multiple subjects can be monitored simultaneously without the need for any other device. However, the drawbacks of such a technology include (1) failure to achieve higher accuracy due to clutter, light fluctuation under different settings, and varied activities in real environments [4]; (2) image-based motion recognition can be require extensive resources and complex processing [3], and (3) it is not cost effective as it requires at least one calibrated camera in each room setting. On the other hand, the pros of body-mounted sensors are that (1) sensed data is independent of environmental conditions and is person-specific; (2) sensors are compact and portable; and (3) processing data does not require a great deal of resources. Alternatively, the downside of body-mounted sensors is service issues in terms of early battery exhaustion. Numerous HMR techniques developed in the past have focused on the acceleration signals in different directions that enable the classification of physical motions [1,2,6]. Motion recognition using multiple sensors instead of relying on a single sensor is also discussed in the literature [6,8,10–13]. However, these approaches are impractical due to the difficulty and inconvenience associated with affixing these sensors to a subject's body and/or clothing. Previous research about the incorporation of multiple sensors for motion detection results in recognition accuracy ranging from 83% to 95% [11]. In [14], the detection accuracy is shown to be less variable for data acquired in the laboratory compared to data collected in a real life environment. The detection accuracy drops from 95% in the laboratory environment to 65% [14] in the real world.

In the literature regarding the features, most studies incorporated fast Fourier transform (FFT) [15], while others used wavelet transform [6,8], support vector machines (SVM), signal-magnitude area (SMA), mean, variance, entropy, and correlations [1,2,5,11]. However, these features are computed using a wide time window, which is not very effective for detecting rapid transitional movements, such as sitting to standing. In [1], short-term transition motions are detected with 95% accuracy.

Methodologies investigated to this point follow heuristic classifiers, Gaussian mixture models (GMM) [16], support vector machines [12], and hidden Markov model (HMM) [17,18]. However, those approaches consider a fixed number of human motions. In other words, most studies follow parametric classification with a known number of motions. In a daily routine, human motions are not fixed—instead they may vary, and new human motion can also occur. Therefore, motion recognition becomes more challenging when the non-parametric nature of human motions is considered. Non-parametric behavior refers to the fact that the number of motions observed may increase as the amount of data increases, and may not remain fixed or could be unknown *a priori*. Therefore, parametric approaches for motion detection fail to achieve higher accuracy when new or transitional motions are observed, such as state transition motions (sitting to standing) for which the motion recognition accuracy is 78% [1,19].

In this paper, we propose motion-dependent sensor metrics to identify human motion. Sensors produce a set of unique signal metrics for various human motions. A signal metric is a component of the sensor readings, such as acceleration in the *x*, *y* and *z* axes of a MEMS accelerometer. These motion-dependent unique signals enable the identification of various motions, including standing, walking, running, taking the stairs, riding an elevator, and more. We considered the autocorrelation function of the tilt angle and the variance of acceleration against the *x* and *y* axes as features for clustering. Our proposed technique differs from the conventional technique in the following ways:

- Selected features are independent of the sensor device, but only dependent of a particular motion.
- It is an observation-based detection system that does not require any protocol or any active coordination among devices.
- Unsupervised clustering is used without any prior knowledge about the number of clusters.

Our contributions in this study are as follows:

- We propose a non-parametric human motion recognition technique that can detect and recognize an unbounded number of motions (clusters). By unbounded, we mean that the number of clusters (activities) is not fixed. Our techniques can automatically cluster those motions without any prior information.
- The accuracy of the motion detection ranges from 97% to 99% with an unknown number of clusters.
- We compute the Kullback–Leibler divergence (KLD) for newly detected motion using already recognized motions. This enables the system to draw inferences regarding the newly detected motions and cluster them.
- The proposed cluster algorithm collects no prior information about the number of motions, and achieves higher accuracy in detecting the number of clusters compared to the conventional method.

The rest of the paper is organized as follows: Section 2 presents current literature describing various motion recognition techniques. Section 3 presents the system model and feature space with an explanation of each feature. Section 4 presents the non-parametric inference and Section 5 explains the non-parametric inference for motion recognition. Section 6 presents the experimental setup, Section 7 demonstrates experimental results, and Section 8 concludes this paper.

## 2. Literature Review

In this section, we review recently developed approaches in the motion recognition domain. Khan *et al.* [19] used autoregressive (AR) modeling of three-dimensional data from the accelerometer. In their work, AR was combined with the signal-magnitude-area and tilt angle obtained from the sensor readings; all of these parameters formed a feature space for the classification of different gaits. These features were utilized to classify a fixed number of human motions, such as standing, walking, running, and lying down. A significant improvement in the detection rate was achieved (95% to 99%); however, the detection rate decreased as new motions were incorporated [1].

Adil *et al.* [1] proposed a system that addressed motion recognition for more than ten gaits with a significant detection rate. Linear discriminant analysis was incorporated in order to extract features. They proposed a hierarchical recognition method for the classification and also explored transitional state change detection. The broad set of motions was divided into three categories: (1) static (stand, sit); (2) transitional (sit-stand, walk-stand, *etc*.); (3) dynamic (run, walk, *etc.*). The achieved accuracy was 97%; however, a fixed number of motions was considered for classification.

Ling *et al.* [11] used multiple sensors (five) for motion recognition. The mean, energy, frequency-domain entropy, and correlation of the data were computed, serving as a source for motion detection. Decision trees outperformed other techniques, attaining an accuracy of 84%. Accelerometers mounted on a subject's thigh and wrists were used to detect their daily motions in a naturalistic way. The fast Fourier transform (FFT) and decision tree algorithm were used to classify different human gaits. Frequency-domain entropy was calculated as the normalized information entropy of the FFT component magnitudes of the signals. The decision tree algorithm was able to recognize the motion style with a higher number of labels, such as walking slowly and walking briskly. However, the overall detection accuracy was 80%.

Rodrigo *et al.* [7] proposed sensor-based human motion recognition using the hidden Markov model (HMM) with a large feature set. Candidate features were obtained from the feature space, while the HMM classifier was used to classify the data points for different motions. The multiple sensor-based approach was used to gather data features, and then only those features that are relevant were utilized for the target classes. Genetic algorithms were incorporated to explore the feature set then to select and utilize only those features that are most relevant to the target motion. However, the accuracy of predicting human motion decreases with an increase in the number of motions to be predicted. This is due to the uncertainty that arises from a greater number of classes. Therefore, the probability of assigning a data point to its correct class decreases.

Slyper *et al.* [8] demonstrated an animation system for e-textile application using multiple MEMS accelerometers that are embedded in a shirt. The accelerometer readings are continuously matched against accelerations computed from existing motion capture data, and an avatar is animated with the closest match using wavelet transform, which is a parametric approach.

Ravi *et al.* [9] formulated human motion recognition as a parametric classification problem. Authors proposed an approach to classify different human motions and selected only those features that are important for classification. The following features were used to classify motions: correlation, mean, standard deviation, and energy. The correlation was calculated between each pair of axes as the ratio of covariance and the product of standard deviation. Motions were classified with reasonable accuracy using the above mentioned features.

Practically, human motions are dynamic in that the number of motions varies with time. For example, a person at a particular moment is standing with friend, then after some time he or she starts walking. He or she may then start cycling and during cycling he or she may fall. The flow of different motions performed by a person can fluctuate over time and vary by individual, making it very likely that a motion recognition system will encounter unfamiliar motions. In other words, the number of motions shows non-parametric behavior. Therefore, a flexible system that can incorporate the time varying behavior of human motions, instead of relying on a fixed number of motions, is needed. In this paper, we propose a non-parametric Bayesian technique that is flexible enough to accommodate the detection and clustering of newly observed motions in order to account for the dynamic and unexpected nature of human motions that vary with time.

System architecture and feature space for the proposed non-parametric human motion recognition will be described in the next section.

## 3. System Architecture and Feature Space for Motion Recognition

**Figure 1.**
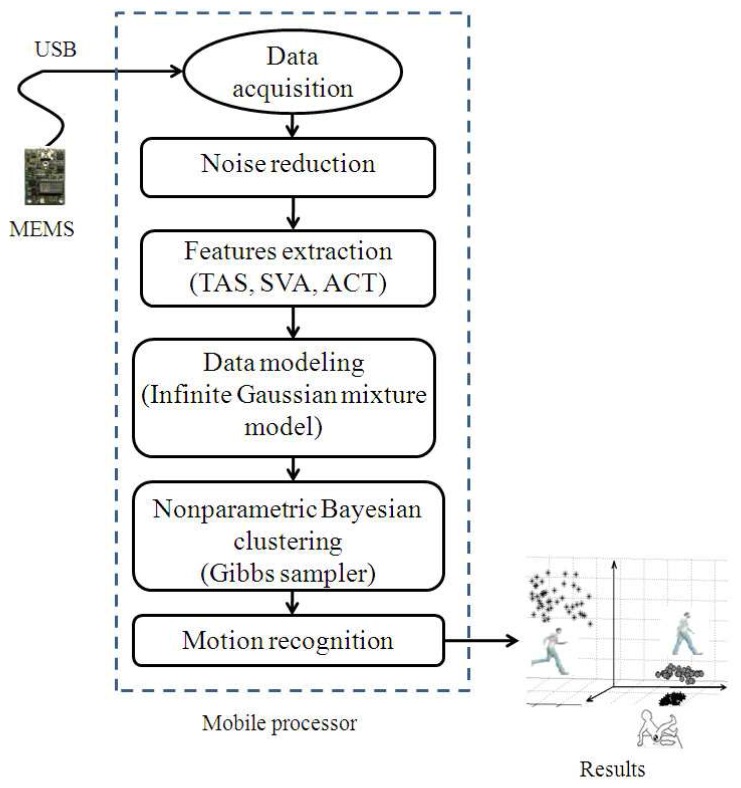
System architecture with components and feature space for the proposed motion recognition.

The architecture of the proposed system for human motion recognition is illustrated in Figure 1. Each rectangle in the system architecture corresponds to a component responsible for performing a particular task. The system architecture is divided into the following components.

While recording real-time data from a MEMS sensor, the output includes noise. It is important to remove this noise before extracting the features from the sensor output. This component tends to remove signal outliers by filtering the signal. The feature space for motion recognition is described in the following subsection, and infinite Gaussian modeling and non-parametric Bayesian inference modeling are discussed in detail in the following sections. The Gibbs sampler results in clusters, where each cluster corresponds to a particular human motion. The clusters obtained from the Gibbs sampler are further mapped to a particular recognized human motion.

**Figure 2.**
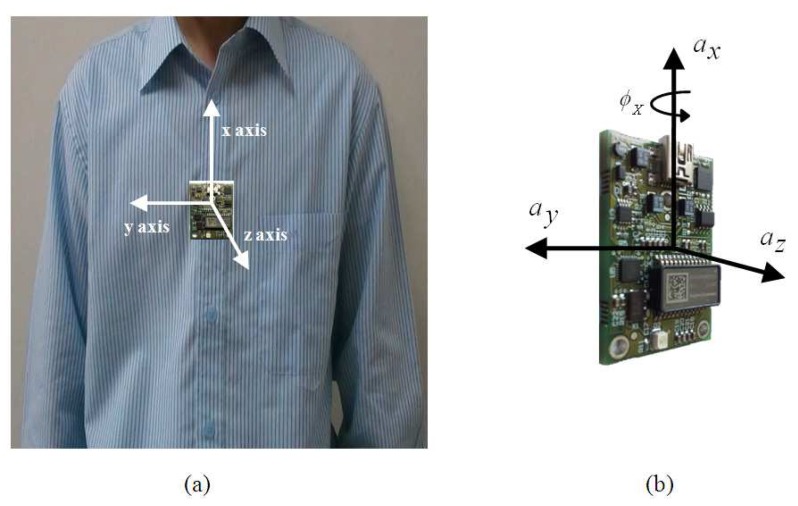
The experimental setup for MEMS sensor: (**a**) A MEMS sensor tagged on the subject's chest; (**b**) Three axes of MEMS accelerometers for experimental setup.

In this section, we introduce the features used in the proposed motion recognition system. To distinguish among different motions, we need to identify unique features that govern various human motions. These features can be extracted from the readings taken by the MEMS sensor attached to the human body. We used an inertial MEMS sensor system (SD777) with a three-axis accelerometer and a single-axis gyroscope. The inertial MEMS sensor (SD777) is a device that measures its own acceleration as a four-dimensional (4D) vector. This 4D vector includes two measurement ranges for the gyroscope, ±100 °/s and ±300 °/s, and one measurement range accelerometer (three axes) from ±1 g to ±5 g. The acceleration can be defined as the rate of change of speed, (*m*/*s*^2^). The acceleration corresponding to each axis can be recorded by a sensor mounted on the subject's chest, as shown in Figure 2.

For data collection, we placed the accelerometer device on the chest of the subject. We obtained a 4D data set from the accelerometer, consisting of the acceleration on three axes and one gyroscope reading. We propose using the following features for human motion recognition: (1) cumulative sum of a gyroscope's angular speed, tilt angle *ϕ*; (2) sum of the variance of the acceleration in the *x* and *y* axes; and (3) the autocorrelation function of the tilt angle, *ϕ*. The selected features are unique to each human motion, and all of the features are dependent on human motion. In this study, we assumed that all of the features follow a Gaussian distribution; however the proposed algorithm is not very sensitive to the exact distribution. Each feature is described in detail in the following subsections.

### 3.1. Tilt Angle Sum (TAS)

The tilt angle refers to the tilt of a body in space. The tilt angle can be defined as the angle between the *x* axis and gravitational vector *g*. Accelerometers are often used to calculate a tilt angle. The angle can be calculated from the acceleration as follows:

(1) ϕ=arcsin(a)

where *a* = [*a_x_*, *a_y_*, *a_z_*] and *a_x_*, *a_y_*, *a_z_* are the accelerations along the *x*, *y*, and *z* axes, respectively. To get an accurate tilt angle, they are often combined with one or more gyros, and a combination of data is used to calculate the angle. Both *x* and *y* tilt angles can be sensed simultaneously using the output of all three axes, as shown below.

(2) $$\phi =\text{arctan}\;\left(\frac{a_{x}}{\sqrt{a_{y}^{2}+a_{z}^{2}}}\right)$$

and

(3) $$\rho =\text{arctan}\;\left(\frac{a_{y}}{\sqrt{a_{x}^{2}+a_{z}^{2}}}\right)$$

The cumulative sum of the tilt angle, *ϕ*, is an important feature that follows a unique behavior for most human motions. The tilt angle shows distinguishing behavior in a static condition (stay, lift-up, lift-down) compared with dynamic (run, walk, fall) human motions. The TAS can be represented as follows:

(4) $$\emptyset (n)=\sum_{i=1}^{W}\left(|\;\phi \;|\right)$$

where *n* is a data point from the set of data points *N*. A data point is the observation recorded from the attached MEMS accelerometer, while *W* is the window size of 512 observations.

### 3.2. The Sum of the Variance of Accelerations (SVA)

Most dynamic gaits (walk, run, fall) produce similar signal amplitude readings for the acceleration in the *x* and *y* directions, but the variance of each distinguishes them from one another. The SVA plays a significant role in distinguishing different motions. To distinguish between the resting state and motion, all three axis reading changes are represented by this feature. In [20], the signal magnitude area (SMA) is used to distinguish between static and dynamic movements; however, we use different version of SMA. We tend to include the variance of all three axes, which separates dynamic and static human motions in an efficient way. Dynamic motions such as walking, running and falling follow similar behavior, while the variance of the acceleration along different axes differs, which helps in motion detection. We define SVA as the signal magnitude area, that is, the sum of the variance of the areas under the moduli of integrals in the time domain. The SVA can be represented as follows:

(5) $$\Psi (n)=\sum_{i=1}^{W}\left(\text{var}(\int_{t}a_{x}(t)\text{dt}\right)+\text{var}\left(\int_{t}a_{y}(t)\text{dt}\right)+\text{var}\left(\int_{t}a_{z}(t)\text{dt}\right))$$

where Ψ(*n*) is the value calculated for a single window. *a_x_*(*t*), *a_y_*(*t*), and *a_z_*(*t*) are the body acceleration components in the *x*-axis, *y*-axis, and *z*-axis samples, respectively. This feature is calculated by summing each sampled value progressively The value obtained from this feature varies for the dynamic and static movements of the human under consideration.

### 3.3. Autocorrelation Coefficient of Tilt Angle (ACT)

The autocorrelation of a signal measures the similarity between observations as a function of the time separation between them. The goal is to identify a repeating pattern in the time-domain signal. Let *ϕ* be a repeatable random process, and *i* be the time point after the start of the process, then *ϕ_i_* is the realization of the process at time stamp *i.* Assume that the process is known to have values for the mean (*μ*) and variance (σ) for all times *i.* The autocorrelation between time *a* and *b* can be defined as:

(6) $$R(\phi_{a},\phi_{b})=\frac{E[(\phi_{b}-\mu_{b})(\phi_{a}-\mu_{a})]}{\sigma_{b}\sigma_{a}}$$

where *E* is the expected value, and the expressions for the autocorrelation are not well defined because the variance may be zero or infinity. However, in our data set, the variance is bounded by a well-defined limit. The above mentioned features play an important role in distinguishing among various static and dynamic motions; however, there are some motions that show low similarity with each other in terms of the time-domain signal for the tilt angle, *ϕ* (e.g., walk, run), but some motions have a smooth curve when the tilt angle is plotted as a function of time (picking up, putting down). Therefore, the ACT is an important metric in capturing this distinct and motion-specific variation. The ACT is represented as follows:

(7) $$\Delta (n)=\sum_{i=2}^{W}\frac{R(\phi_{i},\phi_{i-1})}{W-1}$$

where *i* is the realization of the *ϕ* from the time-domain signal.

**Figure 3.**
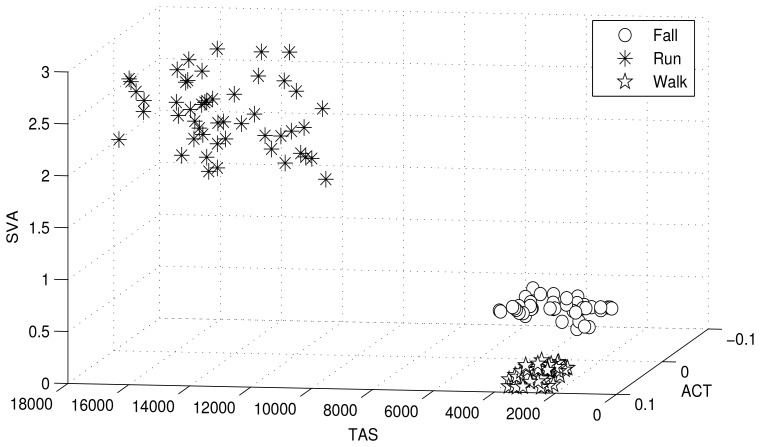
3D feature space with three human motions recognized.

The clusters are modeled as the distribution of a unique hyper-parameter set. Each parameter set represents a unique cluster. The cluster may refer to a particular human motion with a unique hyper-parameter set. Figure 3 illustrates human motion recognition via a 3D feature space, and further demonstrates how the three human motions (walking, running and falling) shown in the figure are dependent on the features used for clustering. Figure 3 shows that the selected features cluster human motions efficiently.

## 4. Non-Parametric Bayesian Inference

Suppose we have *N* data points (observations) constituting all of the above mentioned features, *O⃗* = [*o⃗*_1_, *o⃗*_2_, *o⃗*_3_, *…*, *o⃗_N_*], where each data point is a vector of *D* = 3, representing each feature's value. Our goal is then to build a posterior distribution for the set of observations where the posterior distribution represents the total number of human motions (clusters) in the data set, which can also be used to infer which data point belongs to which motion (cluster). Two generic models exist to represent and model the data points: (a) generative models and (b) discriminant models. We prefer to model our feature space using the generative model [21] rather than a discriminant model for three reasons: (1) discriminant models do not allow one to generate samples from joint distributions of two or more variables; (2) generative models are more flexible in expressing dependencies in a more complex learning environment; and (3) discriminant models are inherently supervised and cannot be extended to unsupervised learning.

The generative model is a model used to randomly generate observable data given hidden parameters. It specifies a joint distribution over observations and labels. Generative models serve two purposes in machine learning: (1) modeling data directly and (2) forming a conditional probability density function. GMM, HMM, naive Bayes (NB), and latent Dirichlet allocation are some examples of generative models. We intend to use the GMM for clustering data points, as the GMM is flexible and can be easily extended to the case in which the number of hidden clusters is unknown. Two models exist in the GMM literature: the finite Gaussian mixture model (FGMM) and the infinite Gaussian mixture model (IGMM). When the number of clusters is known *a priori*, the FGMM is used. On the other hand, if there is no prior knowledge about the number of clusters, then the IGMM can be used. Since we do not limit the number of human motions (clusters) to any fixed number, we intend to use the IGMM rather than the FGMM. However, both models are closely related to each other, so to grasp the idea of the IGMM, an understanding of the FGMM is also necessary. Before explaining the GMMs, we discuss the Dirichlet distribution that governs both models.

### 4.1. Dirichlet Distribution

The Dirichlet distribution is a continuous multivariate distribution parameterized by the vector *α* of the positive real. Furthermore, the multivariate generalization of the beta distribution is also a Dirichlet distribution [22]. The Dirichlet distribution is used as a prior distribution in the Bayes inference engine due to the following two reasons: (1) the Dirichlet distribution is the conjugate prior of the categorical and multinomial distribution; (2) the probability distribution function of the Dirichlet distribution results in the belief about *K* components (clusters) that constitutes the Gaussian mixture model.

The Dirichlet distribution of order K ≥ 2 with parameters *α*_1_, *α*_2_, …, *α_k_* > 0 has a probability density function with respect to the Lebesgue measure on the Euclidean space *R^K^*^−1^ given by:

(8) $$f(o_{1},o_{2},o_{3},\ldots ,o_{K-1})=\frac{1}{B(\alpha )}\prod_{i=1}^{K}o_{i}^{\alpha_{i}-1}$$

for all *o*_1_, *o*_2_, *o*_3_, …, *o_k_*_−1_ > 0 satisfying *o*_1_ + *o*_2_, …, *o_k_*_−1_ < 1 [21,23]. The normalizing constant is a multinomial beta function, which can be expressed in terms of the gamma function:

(9) $$B\;\begin{array}{cc} (\alpha )=\frac{\prod_{i=1}^{K}\Gamma (\alpha_{i})}{\Gamma (\sum_{i=1}^{K}\alpha_{i})}, & \alpha =(\alpha_{1},\alpha_{2},\ldots ,\alpha_{K}) \end{array}$$

### 4.2. Finite Gaussian Mixture Model

A FGMM is a hidden variable probabilistic model based on weighted multivariate Gaussian random variables. The FGMM provides an accurate approximation for multi-modal probability density estimation and clusters data points if the hidden variables are interpreted as class labels. The FGMM assumes that all of the data points are generated from a finite number of Gaussians with unknown parameters.

**Figure 4.**
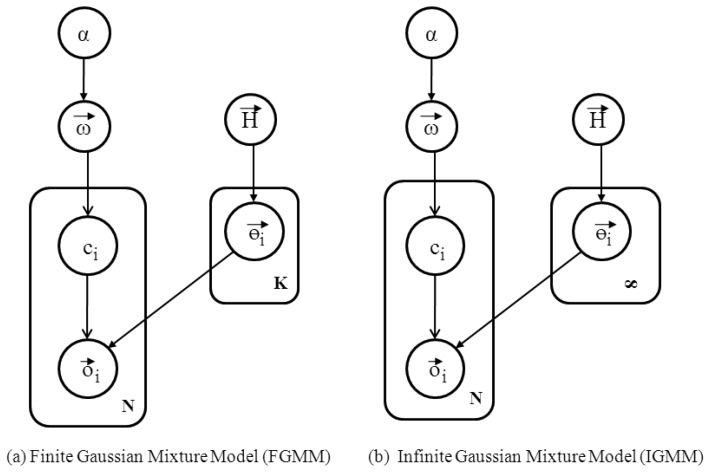
Finite and Infinite Gaussian Mixture Models.

Recall from the previous section that we know matrix *O⃗* = [*o⃗*_1_, *o⃗*_2_, *o⃗*_3_, …, *o⃗_n_*], with *N* number of data points where each *o⃗_i_* represents a vector with *D* dimensions. The number of dimensions *D* is fixed at three in our problem, representing the features space discussed in the previous section. Figure 4 illustrates the finite Gaussian mixture model. Each cluster in the mixture constitutes weight *ω⃗_k_*, which is the probability of assigning a data point to one of cluster (*K*) and *ω⃗ =* [*ω*_1_, *ω*_2_, *ω*_3_, …, *ω_N_*]. Since the Gaussian mixture is a multi-modal probability distribution with a different set of parameters corresponding to each cluster in the mixture, each cluster follows a Gaussian distribution with parameters *θ⃗_k_*, where *θ⃗_k_* is the vector with a mean, *μ⃗_k_* and covariance, Σ*_k_*. Each data point is associated with *c_i_*, indicating to which cluster *K* the data point *o_i_* belongs. The *c_i_* belongs to cluster *k* with probability *ω_k_*. The FGMM can be represented by:

(10) $$\vec{\omega }|\alpha \sim \text{Dir}(\frac{\alpha }{K},\frac{\alpha }{K},\ldots ,\frac{\alpha }{K});{\vec{\theta }}_{k}\sim \vec{H};c_{i}|\vec{\omega }\sim \text{Multinomial}(.|\vec{\omega })$$

(11) $${\vec{x}}_{i}|{\vec{\theta }}_{k}\sim \text{Gaussian}(.|{\vec{\theta }}_{k})$$

The hyper-parameters *H⃗* are the parameters representing prior knowledge, in this case incorporating our prior knowledge about the data points. It realizes the degree of our belief about the underlying system of parameters. They arise when the use of a conjugate prior is necessary to simplify the calculations for posterior estimation. The actual parameters that govern the underlying system are *θ⃗_k_*, while the hyper-parameters tend to accurately estimate the true data. Let *L⃗* be $\left[\frac{\alpha }{K},\frac{\alpha }{K},\cdots ,\frac{\alpha }{K}\right]$.

The FGMM is effective when the number of labels is known, but in reality, we do not know how many clusters are in the mixture. Therefore, we need to have a flexible model that does not have a fixed prior θ⃗. Therefore, θ⃗ follows the base distribution H⃗, which tends to give the model flexibility.

The problem becomes challenging when we do model selection for the FGMM. In our problem, if we have knowledge about the number of human motions, then we could apply the FGMM. However, there is no bound on the number of human motions; they may grow with time. Since it is not appropriate to use the FGMM, the IGMM is introduced in the next subsection for model selection.

### 4.3. Infinite Gaussian Mixture Model

The IGMM is an extension of the FGMM, where *K* → ∞. It is assumed that the number of clusters tends to go to countably infinity because the number of human motions a person can perform is bounded (finite). Therefore, the IGMM can be utilized to model our problem in a generative way for a given data set. Figure 4(b) shows the IGMM as the number of clusters goes to infinity. In the FGMM, the term *ω⃗* is dependent on *K*; as the number of clusters *K* increases, the value of *ω⃗* is affected. It is possible to work on the infinite dimensional model, which will integrate out *ω⃗.* Therefore, *ω⃗* can be marginalized out due to the Dirichlet prior because the Dirichlet prior is the conjugate of the discrete multinomial likelihood. The use of a conjugate prior helps us to integrate our complex integrations. The IGMM can be represented as follows:

(12) $$\vec{\omega }|\alpha \sim \text{Stick}(\alpha );c_{i}|\vec{\omega }\sim \text{Multinomial}(.|\vec{\omega });\vec{\theta }\sim \vec{H};o_{i}|c_{k}{\vec{\theta }}_{k}\sim \text{Gaussion}(.|{\vec{\mu }}_{k},\sum_{k})$$

where *θ⃗_k_* ∼ *H⃗* and the conjugate prior for multivariate normal is inverse Wishart random variable [24].

(13) $$\Sigma_{k}\sim \text{inverseWishort}(\Lambda_{o});{\vec{\mu }}_{k}\sim G({\vec{\mu }}_{o},\frac{\Sigma_{k}}{K_{o}})$$

where the Stick breaking follows beta distribution and is given as:

(14) $$\vec{\omega }\sim \text{Beta}(1,\alpha );\omega_{k}=\omega_{k}^{\prime }\prod_{j=1}^{K-1}\left(1-\omega_{j}^{\prime }\right);K\to \infty$$

where *ω*′ is an initial probability for each cluster. The conjugate prior for the multivariate normal distribution is the inverse Wishart, playing a significant role in the posterior estimation for labels *c_i_*. The prior hyper-parameters for the model are given as *H⃗* = {*μ_o_*, *k_o_*, *υ_o_*, Λ*_o_*}.

Stick breaking process is used in the IGMM to model and imparts flexibility in terms of a variable number of clusters. In the stick breaking process, a stick of unit length is assumed, which can be represented as $\sum_{i=1}^{K}\omega_{i}=1$. The stick breaking process begins by breaking the stick into two parts, modeled using the Beta distribution [24], where the length of one of the parts is *ω*_1_, which corresponds to the weight. The same process is repeated for the remaining part of the stick (1 − *ω*_1_). The countably infinite concept that we discussed in the previous subsection is realized here in the form of the Stick breaking process.

The IGMM fully models the problem under consideration, where the number of human motions is not known. Each type of motion forms a cluster with parameters *θ⃗_k_*. The parameter vector consists of *μ⃗_k_* and Σ*_k_*. The mixing weights (*ω⃗*) are obtained from the Stick breaking process, where *ω⃗_k_* is the probability that a data point belongs to cluster *k*. The *α* represents our confidence in the model parameters. Data modeling is done in this section, and we need a non-parametric clustering approach that clusters the data points based on the prior information. In the next section, we explain how we cluster data into different clusters based on non-parametric Bayesian inference using Gibbs sampling.

## 5. Non-Parametric Bayesian Inference for Motion Recognition

In this section, we focus on the non-parametric Bayesian inference model for motion recognition. We define the labels *C⃗* = [*c*_1_, *c*_2_, …, *c_N_*] for each data point *O⃗*, where *c_i_* indicates the cluster to which the data point *o_i_* belongs. In this section, we answer the following questions: *(1) How many human motions have generated the data set? (2) Which human motion does each data point o⃗_i_ result from?* Hence, the parameter of interest is *C⃗*; once we find *C⃗*, then we are able to answer the above questions.

The IGMM is a generic model that can be extended by numerous approaches for parameter estimation described in the literature. For example, for parameter estimation, the following methods can be utilized with the IGMM for clustering: expectation maximization (EM), Markov chain Monte Carlo (MCMC), moment matching, spectral methods, *etc.* In our problem, we restrict ourselves to the use of MCMC due to its clustering accuracy. The realization of MCMC is the Gibbs sampling algorithm [25] that we incorporate to draw inferences about the data points. In the Gibbs sampling approach, parameters are integrated out, which results in lower complexity. The Gibbs sampler is used to obtain a sequence of random samples from a joint probability distribution of more than one random variable. The random samples obtained can be used to approximate the joint probability distribution, to approximate unknown parameters, or to compute integrals [25].

Recall that we are interested in the *C⃗*, which is difficult to find due to the complex integration process. Therefore, instead of finding the joint probability distribution, we tend to calculate *P*(*c_i_* = *k*|*O⃗*, *C⃗*_−1_, *α; H⃗*). Therefore, applying the Bayes rule, we get:

(15) $$P(c_{i}=k|\vec{O},{\vec{C}}_{-i},\alpha ;\vec{H})=P(c_{i}=k|{\vec{O}}_{-i},\alpha ,{\vec{\theta }}_{k},\vec{H},{\vec{o}}_{i})$$

(16) $$P(c_{i}=k|\vec{O},{\vec{C}}_{-i},\alpha ;\vec{H})\propto P(o_{i}|{\vec{O}}_{-i};\vec{H})P(c_{i}=k|{\vec{C}}_{-i},\alpha )$$

where ∼ is introduced to replace the normalization constant from the above equation. In the equation above, the term *H⃗* is a Student t-distribution, which can be computed easily. The Student t-distribution is used to compute the mean of a normally distributed population where the sample size is small and the population standard deviation is unknown [24]. The term *P*(*c_i_* = *k*|*C⃗*_−_*_i_*, *α*) in the equation is unknown, and will be calculated in the following subsection.

### 5.1. FGMM and Motion Recognition

In the context of FGMM, the number of clusters are fixed and assumed to be *K.* The value of interest here is *P*(*c_i_* = *k*|*C⃗_−i_*, *α*) under *K* clusters. The FGMM is modeled in Equation (10). Let us assume that *N* data points are clustered and we received *o⃗_N_*_+1_, then the probability of assigning this new data point to a cluster *k* is given below:

(17) $$P(c_{N+1}=k|c_{1‥N};\alpha ,\vec{L})=\int P(c_{N+1}=k|\vec{\omega })P(\vec{\omega }|c_{1:N};\alpha ,\vec{L})d\vec{\omega }$$

(18) $$=\int P(c_{N+1}=k|\vec{\omega })P(\vec{\omega };\alpha *,\vec{L}*)d\vec{\omega }$$

(19) $$=E(P(c_{N+1}=k|\vec{\omega }))$$

(20) $$=\frac{\alpha \;*\;m\;*}{\sum_{i=1}^{K}\alpha \;*\;m_{i}^{*}}=m_{k}^{*}.$$

Equation (17) is the marginal distribution, where *ω⃗* is integrated out. Equation (19) is the expected value of *ω⃗*. Equation (20) gives the marginal probability of allocating a new data point to the clusters already present, where $m_{k}=\frac{\alpha }{K}$ is the number of data points in the cluster *k.* The posterior distribution of the weights is the Dirichlet distribution with updated prior parameters [25],

(21) $$\alpha *=\alpha +N;\vec{L}*=\frac{\alpha \vec{L}+\text{NF}}{\alpha +N}$$

where *F* is empirical distribution. The cluster assignment for the *o_N_*_+1_ can be given by [21,23]:

(22) $$P(c_{N+1}=k|c_{1‥N};\alpha ,\vec{L})=\frac{\alpha /K+n_{k}}{\alpha +N}$$

where *n_k_* is the number of data points originating from the same cluster.

### 5.2. IGMM and Motion Recognition

Now, we will relax the limit over the *K* and let *K* → ∞. In this section, we extend the model from finite *K* to infinity, therefore, when *K →* ∞ the FGMM transforms to the IGMM. From [21,23], we know that,

(23) $$P(c_{N+1}=k|c_{1‥N};\alpha ,\vec{L})=\frac{n_{k}}{\alpha +N}$$

where the right side of Equation (23) is the probability that data point *N* + 1 belongs to cluster *k.* However, from the property of exchangeability of the non-parametric Bayesian [24], we can replace *N* + 1 with any value of *i* without changing the joint probability. Therefore, we have

(24) $$P(c_{i}=k|{\vec{C}}_{-i};\alpha )=\frac{n_{k,-i}}{\alpha +N-1}$$

where *n_k_*_,−_*_i_* is the number of data points assigned to the *k*th cluster. However, the left side of Equation (24) represents the probability of assigning a data point to an existing cluster (human motion). However, there is also the possibility that new human motion is detected and, as discussed in previous sections, that new human motion detection will follow different feature values. Therefore, we can model the new motion detection with the probability given by:

(25) $$P(c_{i}\neq c_{j},\forall j\neq i|{\vec{C}}_{-i},\alpha )=1-\frac{\sum_{j=1}^{K}n_{j,-i}}{\alpha +N-1}=\frac{\alpha }{\alpha +N-1}$$

From Equation (25), we can see that probability of assigning a data point to a new cluster is equal to 1 minus the sum of all probabilities assigned to existing clusters. This process is called Chinese restaurant process (CRP) [26]. In the CRP the number of tables (clusters) and the number of customers sitting at a particular table (data points) can be infinity. The first customer arriving at the restaurant will always sit at the first table. However, the second customer arriving at the restaurant will want to sit at the first table or he may choose a new table, and this process continues for the rest of the customers arriving at the restaurant. The probability that a customer will sit at an already occupied table depends on the number of customers already seated at the table *m_k_*; given in Equation (24). However, the probability of sitting at a new table is proportional to *α*; given in Equation (25).

At this stage, we need Gibbs sampler to obtain the samples of *C⃗*. After observing *N* − 1 data points, we can update priors using Bayes rule. Therefore, the probability of assigning a data point to the represented cluster is given as:

(26) $$P(c_{i}=k|{\vec{C}}_{-i},\alpha ,\vec{\theta },\vec{H},\vec{O})=P(c_{i}=k|{\vec{C}}_{-i},\alpha )P({\vec{o}}_{i}|{\vec{\theta }}_{k})$$

where *C⃗*_−_*_i_* is the set of observations currently assigned to cluster *i* except *o_i_*, *P*(*c_i_ = k*|*C*_−_*_i_*, *α*) in the Equation (26) follows the Chinese restaurant process and *P*(*o⃗_i_*|*θ⃗_k_*) follows Gaussian with parameters *θ⃗_k_*. The probability that a data point belongs to a represented cluster is given by:

(27) $$P(c_{i}\neq j,\forall j\neq i|{\vec{C}}_{-i},\alpha ,\vec{\theta },\vec{H},\vec{O})=\frac{\alpha }{\alpha +N-1}\int_{\vec{\theta }}P({\vec{o}}_{i}|\vec{\theta })P(\vec{\theta }|\vec{H})d\vec{\theta }$$

The integration is the marginal probability, where *θ⃗* is integrated out. The use of the conjugate prior has made the integration tractable. Moreover, we know that the conjugate prior of the multivariate normal distribution is the inverse Wishart. By integrating out the parameter *θ⃗* the sample size of the problem is reduced and the Gibbs sampler will converge early. Therefore, we adopt collapsed Gibbs sampler [27] to increase the performance in terms of reduced complexity.

### 5.3. Collapsed Gibbs Sampler

Remember that our goal is to estimate the posterior distribution for the infinite Gaussian mixture model using the Gibbs sampler. Therefore, we have already chosen the inverse Wishart distribution which make it possible integrate out parameters *θ⃗_k_*. In the collapsed Gibbs sampling approach, the parameter *θ⃗_k_* is integrated out. Therefore, it is possible to obtain a closed-form solution for the posterior distribution. From [23], we have,

(28) $$P(c_{i}=k|{\vec{C}}_{-i},\alpha ,\vec{H},\vec{O})=P(c_{i}=k|{\vec{O}}_{-i},\alpha )P({\vec{o}}_{i}|{\vec{O}}_{k,-i};\vec{H})$$

The first term on the right side of Equation (28) follows the Chinese restaurant process and is given by Equation (23). However, the second term follows the multivariate Student-t [24]. The hyper-parameters *H⃗* is used to simplify the integration steps in the posterior estimation. Therefore, we choose the inverse Wishart as a prior for the Σ*_k_* distribution and Gaussian distribution for *μ⃗_k_*.

(29) $$\sum_{k}\sim {\text{inverseWishart}}_{\upsilon_{o}}(\Lambda_{o}^{-1})$$

(30) $${\vec{\mu }}_{k}\sim \text{Gaussian}({\vec{\mu }}_{o},\sum_{k}/k_{o})$$

From [24], we can write the second term in the Equation (28) as,

(31) $$P({\vec{o}}_{i}|{\vec{O}}_{k,-i};\vec{H})\sim t_{\upsilon_{n}}-D+1\left[{\vec{\mu }}_{n},\frac{\Lambda_{n},(k_{n}+1)}{k_{n}(\upsilon_{n}-D+1)}\right]$$

where

(32) $${\vec{\mu }}_{n}=\frac{k_{o}}{k_{o}+N}{\vec{\mu }}_{o}+\frac{N}{k_{o}+N}\vec{O}$$

(33) $$k_{n}=k_{o}+N,\upsilon_{n}=\upsilon_{o}+N$$

(34) $$\Lambda_{n}=\Lambda_{o}+S+\frac{k_{o}n}{k_{o}+N}((\vec{O})-{\vec{\mu }}_{o}){\left(\vec{O}-{\vec{\mu }}_{o}\right)}^{T}$$

(35) $$\vec{O}=({\vec{o}}_{1},{\vec{o}}_{2},\ldots ,{\vec{o}}_{N})/N$$

where *D* is the number of dimension, *S* is the sample variance of the observations *O⃗*, and *μ⃗_n_*, *κ_n_*, *υ_n_*, Λ*_n_* are the updated hyper-parameters. *n* is the number of observations in that particular cluster. The advantage of integrating out the parameters is that it reduces the sample space, resulting in a quick convergence [26]. Using Equation (31), the distribution can be determined as a multivariate Student-t distribution with hyper-parameters,

(36) $$P({\vec{o}}_{i};\vec{H})\sim t_{\upsilon_{n}}-D+1\left[{\vec{\mu }}_{n},\frac{\Lambda_{n},(k_{n}+1)}{k_{n}(\upsilon_{n}-D+1)}\right]$$

The above results in two cases for clustering. One is the case when data point belongs to one of the represented cluster from *K* clusters. In the second case, the data point belongs to a new cluster. The first case can be represented as,

(37) $$P(c_{i}=k|{\vec{C}}_{-i},\vec{O};\alpha ,\vec{H})\sim \frac{m_{k,-i}}{\alpha +N-1}t_{\upsilon_{n}}-D+1\left[{\vec{\mu }}_{n},\frac{\Lambda_{n},(k_{n}+1)}{k_{n}(\upsilon_{n}-D+1)}\right]$$

In the second case when the data point observed is assigned a new cluster, it can be represented as,

(38) $$P(c_{i}\neq j,\forall j\neq i|{\vec{C}}_{-i},\vec{O},\alpha ,\vec{H})\sim \frac{\alpha }{\alpha +N-1}t_{\upsilon_{n}}-D+1\left[{\vec{\mu }}_{n},\frac{\Lambda_{n},(k_{n}+1)}{k_{n}(\upsilon_{n}-D+1)}\right]$$

The posterior derived is used in the collapsed Gibbs sampler.


**Algorithm 1**: Non-parametric Bayesian clustering algorithm for human motion recognition using collapsed Gibbs sampler
**Input**: *O⃗*(*o⃗_i_, D*), Sweeps, Γ(*a*, *b*), *H⃗***Output**: *K*, *c_i_***1****begin****2** *C⃗←c*_1_, *c*_2_, …,*c_Ḱ_***3** *K_+_←*0;**4** **foreach**
*s in Sweeps*
**do****5**  C_s_ ← *C_s_*_−1_**6**  **foreach**
*s in Sweeps*
**do****7**   
$m_{-i}\leftarrow \sum_{j=1}^{Ḱ}\left(c_{j}==c_{i}\right)-1;$**8**   **if**
*m*_−_*_i_* == 0 **then****9**    *c_j_*←*c_j_*-1; ∀j ≻ i**10**    *K_+_* ← *K_+_* -1**11**   **end****12**   dim ← length(*μ_D_*)**13**   **foreach**
*i in K*
**do****14**    covariances(i,dim)← iWishart(λ, v)**15**    means← MVrnd(*μ*, covariances(i,dim))**16**    
$m_{k}=\sum_{i=1}^{R}\text{I}\left(c_{i}=k\right)$**17**    *c_i_* ← MVrnd(means,covariances)**18**   **end****19**   /* Estimate the prior *P(o⃗_i_|O⃗ H⃗*) using Equation (31) */**20**   **if**
*c_i_* ⋏ *K_+_*
**then****21**    *K_+_* ← *K_+_+*1**22**   **end****23**   /* Estimate the prior *P*(*c_i_* = *k*|C⃗*_−i_*, *α*) using Equations (23) and (24) */**24**  **end****25** **end****26** /* Collapsed Gibbs Sampling */**27** /* Estimate the posterior *P*(*c_i_ = k*|*O⃗*, *C⃗_−i_*, *α*; *H⃗*) using Equation (37) */**28****29****end**


In Algorithm 1, input parameters are the measured data point from MEMS sensor, the number of sweep to monitor the convergence of algorithm, and hyper-parameters. The data model is estimated using the observed data and hyper-parameters. Algorithm 1 runs for the specified number of sweeps (steps) till convergence after initialization procedure. For example, the loop in the step 6 runs for 100 sweeps. The samples obtained for each data point from the conditional distribution are used to estimate the joint distribution of all variables. In Algorithm 1, priors are estimated and updated for each step in the Gibbs sampler. After repeated estimation and update process, the final posterior estimation can be obtained at the last step in Algorithm 1.

### 5.4. Motion Recognition Method

The result we get from the collapsed Gibbs sampler is the indicators *C⃗*, and the indicators are associated with every data point that is input into the collapsed Gibbs sampler. The indicator *C⃗* obtained as an output from the collapsed Gibbs sampler answers the two questions that we are supposed to answer. Recall from Section 5, the questions we have to answer are (1) how many clusters represent the data set and (2) which data point belongs to which cluster. As every data point is associated with its respective indicator (*c_i_*, *i*= 1 : *N*), the indicator demonstrates to what cluster the data point (*o⃗_i_*, *i* = 1 : *N*) belongs. The first question can be answered by counting the unique values of *c_i_*. The last step in motion recognition is motion mapping. The mean and variance of each cluster can be used to identify human motion. We assume that we are given each motion-dependent value for the feature space. We compare those with the values obtained from the collapsed Gibbs sampler, and the similarity between the two distributions is measured via the Kullback-Leibler divergence. The KLD value is computed between each cluster obtained from the non-parametric Bayesian and the reference training data. The decision is made over a minimum value of KLD.

## 6. Experimental Setup

**Figure 5.**
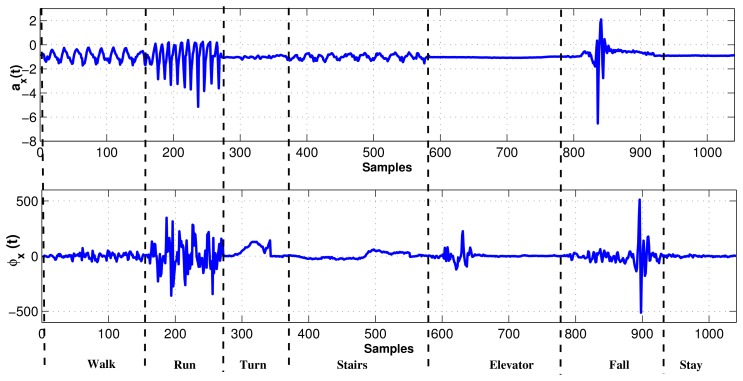
The acceleration (*a_x_*) and tilt angle (*ϕ_x_*) signals of human motions obtained from the MEMS sensor.

**Figure 6.**
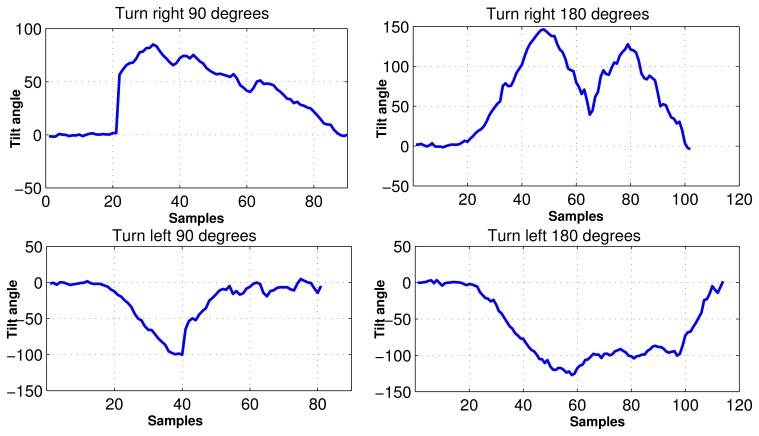
Tilt angle signals for turning motions.

In experiment, the MEMS accelerometer device was mounted to the chest of the individual as shown in Figure 2. The data set for our experiments was collected in an unsupervised way. Every motion was performed by fifty different individuals without any supervision. In consequence, fifty data sets are acquired for each motion, where the distance for walk and run was 10 meters. Data sets were acquired using USB (Universal Serial Bus) interface from the MEMS device. The sampling rate for data acquisition was 45 Hz/sample. We considered human motions such as standing, walking, running, turning, falling, taking the stairs, and taking the elevator, all of which can happen in a building environment. The acceleration (*a_x_*) and tilt angle (*ϕ_x_*) in *x* axis are depicted for different human motions in Figure 5. It is evident from Figure 5 that different motions show different patterns. In Figure 6, the plots for tilt angle are shown for left and right turn. It is clear from the plots in Figure 6 that the left and right turns show distinguishing characteristics for tilt angle. The tilt angle is a useful feature to cluster human motions.

### 6.1. Hyper-Parameters

In this section, we will explain the hyper-parameters that are useful in carrying out the clustering procedure. Since we rely on the IGMM, we have to set the hyper-parameters in such a way that represents our actual true data set. The complexity of the Gibbs sampler grows significantly as the number of data points increases [21,23]. Therefore, it is desirable to set hyper-parameters such that the true data is obtained faster in order to reduce the Gibbs sampler complexity.

Since we have employed the generative model, we have to setup the following hyper-parameters, *H⃗* = {*μ⃗_o_*, *κ_o_*, Λ*_o_*, *υ_o_*} and *α*, where *α* is the concentration parameter that encodes the number of clusters in the data set. As the value of *α* increases, the number of clusters also increases, which is evident from Equation (25). In our scenario we keep the value of *α* small initially because the number of human motions in the initial stage is assumed to be smaller.

The mean vector *μ⃗_o_* of a cluster follows a Gaussian distribution with mean *μ⃗_o_* and covariance Σ*_o_*/*κ_o_*. Therefore, the two hyper-parameters *μ⃗_o_* and *κ_o_* represent the mean for the cluster. Where *κ_o_* is the dispersion measure of the cluster, the higher is the value of *κ_o_*, the closer the clusters are to each other, and vice versa.

The covariance matrix of the cluster depends on the following two hyper-parameters, *υ_o_* and Λ*_o_*. These parameters are related to the inverse Wishart distribution. Parameter *υ_o_* is the degree of freedom while Λ*_o_* quantifies the variability around the mean of the feature space. Suppose that feature point TAS shows a wide range of variability from its mean value, which is specific to a particular human motion such as running. Therefore, this variability is quantified by Λ*_o_*. The value of *υ_o_* represents our confidence about Λ*_o_*.

### 6.2. Mapping Cluster to Motion

The Gibbs sampler results in a number of clusters that constitute the data set, as well as each data point's association with a particular cluster. We assume that the mean values of the features are available for all of the human motions. After clustering, we compare and then map the clusters to the human motion.

## 7. Experimental Results

In this section, we present the performance analysis of the proposed motion detection technique, in addition to comparing our proposed technique with the parametric Fuzzy C-Means (FCM) [28], the unsupervised K-Means clustering algorithm [29], the non-parametric mean-shift clustering method [30]. In the performance analysis, we study the effect of varying the number of human motions. The results demonstrate the effect of introducing new human motion on the clustering accuracy. In the following subsection, we briefly discuss the K-means and mean-shift clustering approaches.

### 7.1. K-means Clustering

In the K-means clustering algorithm, the *N* data points are partitioned into *k* clusters, where each observation belongs to the cluster with the nearest mean. Given a set of observations *o⃗*_1_, *o⃗*_2_, …, *o⃗_N_*, where each observation *o⃗_i_* is a *D*-dimensional vector, the K-means clustering algorithm tends to cluster those *N* observations into *K* sets, where (*K ≤ N*) and *K =* [*k*_1_, *k*_2_, …, *k_K_*], in order to minimize the intra-cluster sum of squares.

(39) $$\text{arg}\min\sum_{i=1}^{K}\sum_{{\vec{o}}_{j}\in k_{i}}{\left\|{\vec{o}}_{j}-{\vec{\mu }}_{i}\right\|}^{2}$$

where *μ⃗_i_* is the mean of points in *k_i_*.

The overall algorithm proceeds in two steps. Initially it is assumed that the initial means for the *K* clusters are given *by* $h_{1}^{1},h_{2}^{1},\ldots ,h_{K}^{1}$, where the two steps for the algorithm are the assignment step and the update step. In the assignment step each observation is assigned to a cluster with minimum mean, represented as,

(40) $$k_{i}^{\left(t\right)}=\left\{{\vec{o}}_{p}:\left\|{\vec{o}}_{p}-{\vec{\mu }}_{i}^{\left(t\right)}\right\|\leq \left\|{\vec{o}}_{p}-{\vec{\mu }}_{j}^{\left(t\right)}\right\|\forall 1\leq j\leq k\right\}$$

where each *o⃗_p_* goes to one of the $k_{i}^{\left(t\right)}$. In the update step new means are calculated based on the observations.

(41) $$h_{i}^{\left(t+1\right)}=\frac{1}{\left|k_{i}^{\left(t\right)}\right|}\sum_{{\vec{o}}_{j}\in k_{i}^{\left(t\right)}}{\vec{o}}_{j}$$

The algorithm converges when there are no updates. The k-means in *D*-dimensions is NP hard.

### 7.2. Mean-Shift Clustering

Mean-shift is a non-parametric clustering algorithm [30]. The mean-shift procedure is obtained by successive computation of the means-shift vector *θ⃗_i_* and translation of the kernel *G*(*o⃗_j_*) by *θ⃗_i_*. The mean-shift procedure is guaranteed to converge at a nearby point where the kernel estimate has zero gradient [30]. It is useful for detecting modes of the density function, which is iterative method with initial start. Here we use Gaussian kernel function for the distance to the current estimate given as, *G*(*o⃗_j_* − *μ⃗_i_*) = $\exp{\left(c\left\|{\vec{o}}_{j}-{\vec{\mu }}_{i}\right\|\right)}^{2}$. The weighted mean of the density for cluster *k* is given as,

(42) $${\vec{\theta }}_{i}=\frac{\sum_{{\vec{o}}_{i}\in N({\vec{o}}_{i})}\exp^{{\left(c\left\|{\vec{o}}_{j}-{\vec{\mu }}_{i}\right\|\right)}^{2}}{\vec{o}}_{i}}{\sum_{{\vec{o}}_{i}\in N({\vec{o}}_{i})}\exp^{{\left(c\left\|{\vec{o}}_{j}-{\vec{\mu }}_{i}\right\|\right)}^{2}}}-{\vec{o}}_{j}$$

where *N*(*o⃗_i_*) is a set of data points for which *G*(*o⃗_i_*) *≠* 0. Similarly, assignment is done *o⃗_i_ = θ⃗_i_* in iterative way until the convergence point is reached.

### 7.3. Performance Evaluation Criteria

The accuracy of the proposed non-parametric Bayesian inference can be evaluated using the following three metrics:

1. The hit rate for detecting the right number of human motions in the data set. The percentage is calculated for the correct number of human motions detected over the total number of trials performed.
2. The hit rate for each data point is realized by assigning every feature point to its correct cluster. It is the percentage of feature points assigned to its correct cluster over the total number of feature points.
3. The false alarm rate can be computed by counting the data points that are assigned to incorrect clusters.

### 7.4. Unforeseen Motion Detection with Limited Data

In this subsection, we will show the efficacy of the proposed motion detection method against unforeseen motions. As discussed in the previous section, human motions vary with time and encountering new motions is anticipated. Moreover, the proposed HMR approach is robust in terms of new motion detection and recognition. For example, a person is walking and instantly falls due to some unavoidable cause. The person's fall is a new event that should be detected and recognized precisely. Therefore, we compared our proposed approach with a parametric Fuzzy C-Means clustering algorithm [28]. In Fuzzy clustering, the data point can belong to more than a single cluster, and a set of membership levels can be associated with each data point based on the strength of a data point's association with a particular cluster. In FCM, each data point *o⃗_i_* has a coefficient that represents its association with a particular cluster *ω⃗_k_*(*o⃗_i_*). In FCM, the centroid of a cluster of the mean of all data points is a weighted measure belonging to the cluster. This can be represented as

(43) $$Q_{k}=\frac{\sum_{\vec{o}}\omega_{k}(\vec{o})\vec{o}}{\sum_{\vec{o}}\omega_{k}(\vec{o})}$$

The algorithm also tend to minimize intra-cluster variance.

**Figure 7.**
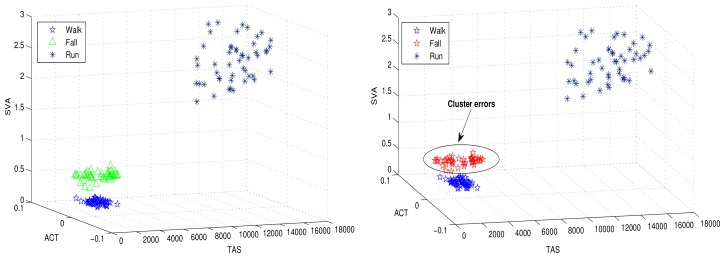
Unforeseen motion detection results by proposed (**left**) and FCM clustering algorithm (**right**).

The accuracies of the proposed and FCM approaches are given in Figure 7. It is evident from the Figure 7 that the FCM clustering approach fails to recognize the new motion (fall) due to its parametric clustering procedure. However, the proposed technique accurately identifies the new motion with 100% accuracy. The performance evaluation is shown in Table 1. In the FCM approach, the new motion is not detected and all of the data points are classified into two clusters (motions). However, the proposed HMR detects the unanticipated fall and clusters with 99.33% accuracy. For the performance metric 1, the proposed approach clusters the data points into three clusters with 100% accuracy, where the PCM clusters the same data points into two clusters.

### 7.5. Recognition Results for Numerous Motions

In this subsection, we show the recognition accuracies for routine daily motions. We compare the proposed HMR technique with the K-means, and observe the performance gains in terms of the performance metric criteria discussed above. Since we used a generative model, we need to set the hyper-parameters in such a way that the hit rate is maximized with a minimum number of errors. Note that the clustering results are highly sensitive to the hyper-parameters. Therefore, the hyper-parameters must be set carefully in order to reduce the chance of cluster errors. We set the values of the hyper-parameters as *H⃗* = {Λ*_o_*, *μ⃗_o_*, *κ_o_*, *υ_o_*}, and *κ_o_* = 0.1, *υ* = 4, Λ = *diag*(0.3), and Γ(3, 2).

### 7.6. Varying Number of Human Motions

**Figure 8.**
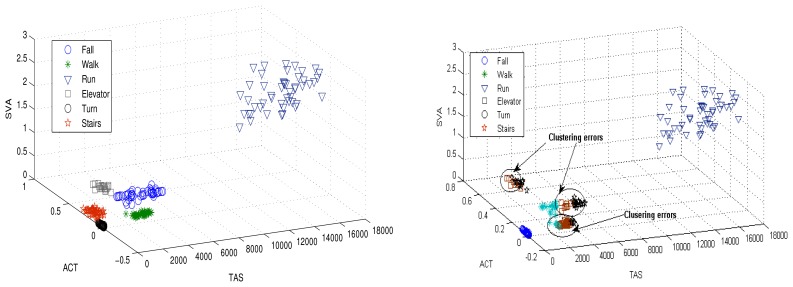
Clustered results for the proposed algorithm (**left**) and the K-Means method (**right**) with six human motions.

In the simulations, we compared the proposed approach with the K-Means clustering algorithm. Figure 8 shows the clustering results for the proposed human motion approach and K-Means clustering. The clustering errors produced by the K-Means are shown in Figure 8. The proposed approach efficiently clustered the human motions.

**Figure 9.**
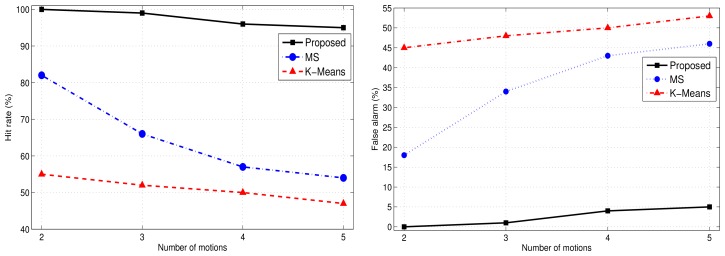
Hit rate (**left**) and false alarm (**right**) results for the proposed algorithm, the K-Means method, and the mean-shift method with varying number of motions.

Figure 9 demonstrates the accuracy and clustering errors for the proposed method, K-Means, and mean-shift clustering approaches. The Type 2 hit rate of the proposed approach outperforms that of the K-Means and mean-shift approaches in terms of accurate data point clustering. The unsupervised K-Means and mean-shift algorithms suffer a significant loss in accuracy.

### 7.7. Kullback–Leibler Divergence and Clustering Accuracy

In this section, we compared the accuracy of the proposed approach with that of the K-Means approach with a varying Kullback–Leibler divergence value. The Kullback–Leibler divergence is the measure of the difference between two probability distributions. Equation (44) represents the measure of the KLD, where D is the number of dimensions in the data set.

(44) $$\text{KLD}=\frac{1}{2}[\text{trace}(\Sigma_{2}^{-1}\Sigma_{1})+({\vec{\mu }}_{2}-{\vec{\mu }}_{1}){-}^{T}\Sigma_{2}^{-1}({\vec{\mu }}_{2}-{\vec{\mu }}_{1})-\ln(\frac{\text{det}\sum_{1}}{\text{det}\sum_{2}}-D)]$$

**Figure 10.**
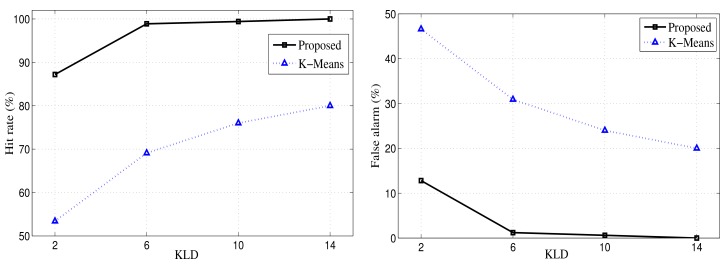
Clustering accuracy (**left**, hit rate; **right**, false alarm) with KLD variations for the proposed algorithm and the K-Means method.

In this section, we show how the clustering results are affected by varying the KLD for the proposed and K-Means clustering approaches. In Figure 10, it is shown that the proposed approach outperforms the K-Means algorithm in terms of its hit rate and false alarm. It is evident from Figure 10 that the hit accuracy of the proposed approach is increased to 100% when the KLD increases, but the K-Means algorithm fails to achieve such high hit rate. The proposed approach attains 100% accuracy at KLD=14 for the clustering data points as shown in Figure 10.

### 7.8. Convergence

**Figure 11.**
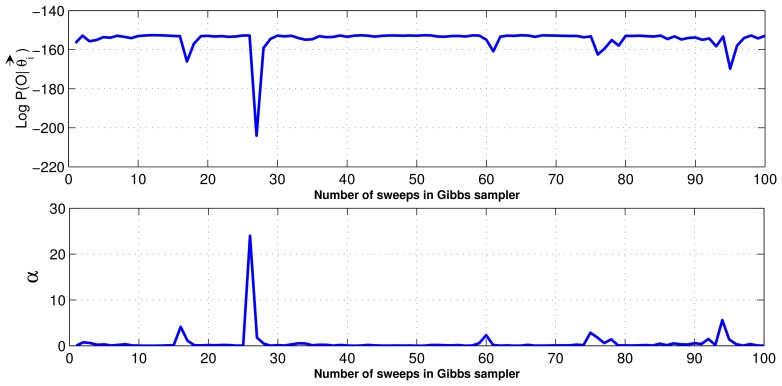
Convergence characteristics of the collapsed Gibbs sampler.

From the simulation results, it is apparent that the proposed collapsed Gibbs sampler converges to a stable state after a few iterations. Figure 11 demonstrates the convergence rate of the collapsed Gibbs sampler. The plot of the random variables are generated, for the log probability of training data under the model and the distribution over the Dirichlet hyper-parameter *α*, in Figure 11. The two graphs are useful in diagnosing the convergence of the Gibbs sampler. Even though the Gibbs sampler converges [31], it is difficult to diagnose the convergence of Gibbs sampler in practice; therefore, visual graphs can help to decide on the convergence. It is clear that the convergence is attained after 30 iterations for the given data set as shown in Figure 11.

## 8. Conclusions

In this study, we proposed a non-parametric Bayesian approach for detecting and clustering various human motions. The proposed work exploits the motion-dependent signal features to model an available data set using the infinite Gaussian mixture model. The collapsed Gibbs sampler is utilized to classify the available data set into various human motions. The experimental results show that the proposed human motion recognition approach significantly outperforms methods including the Fuzzy-C Means, K-Means, and mean-shift approaches. The unsupervised nature of the proposed scheme relaxes the upper bound for the number of human motions under consideration. Therefore, the proposed approach can be extended to many other applications in which the number of underlying clusters is unknown.

## Figures and Tables

**Table 1.** Performance metrics results for FCM and proposed HMR.

**Motion**	**FCM**		**Proposed HMR**	

	**Type 2: Hit Accuracy**	**Type 3: False Alarm**	**Type 2: Hit Accuracy**	**Type 3: False Alarm**

	(**%**)	(**%**)	(**%**)	(**%**)
Walk	100	66.67	100	0
Run	100	0	100	0
Fall (new)	0	100	99.33	0.67
